# Recent Advances in Wearable Biosensors for Human Health Monitoring

**DOI:** 10.3390/bios16010027

**Published:** 2026-01-01

**Authors:** Xiaojun Xian

**Affiliations:** The McComish Department of Electrical Engineering and Computer Science, Jerome J. Lohr College of Engineering, South Dakota State University, Brookings, SD 57007, USA; xiaojun.xian@sdstate.edu

As the healthcare system transitions from traditional hospital-centered care to point-of-care and home-care systems, the demand for wearable sensors is increasing rapidly due to their ability to continuously monitor human health and support medical diagnostics [1,2,3]. There has been substantial progress in the development of wearable sensors for detecting biomarkers in various biofluids such as sweat [4], tears [5], and interstitial fluid [6], tracking volatile biomarkers in human breath [7,8] and transcutaneous gases [9,10], and monitoring physical physiological signals including heart rate [11], blood pressure [12], body temperature [13], and ECG [14].

In the Special Issue “Recent Advances in Wearable Biosensors for Human Health Monitoring”, five review articles and three original research papers provide a broad perspective covering breath analysis, transcutaneous and sweat/tear biomarker detection, cardiovascular monitoring, autism spectrum disorder management, physical rehabilitation, and wearable antenna development. By coupling wearable sensors with advances in materials, chemistry, biology, and mechanics, interdisciplinary collaborations accelerate the development of wearable biosensors for health monitoring.

Non-invasive methods carry a low risk of infection and allow fast and easier operation. Among the non-invasive diagnostic approaches, detecting volatile biomarkers in human breath and transcutaneous gases shows great potential for convenient disease management [15]. Diabetes is a significant public health challenge, with high prevalence among people aged 20–79 years [16]. Frequent monitoring of blood glucose in diabetic patients is essential to ensure their blood glucose level remains within the normal physiological range [17]. Rather than relying on invasive blood glucose measurements, breath ketone detection offers a simple, non-invasive alternative for diabetes diagnosis and monitoring. In contribution 1, Marfatia et al., reviewed recent breath ketone analysis technologies in Type 1 Diabetes, evaluated their accuracy and sensitivity, and investigated the correlation between breath acetone (BrAce) levels and blood ketones. This review has demonstrated that nanomaterial-based sensing shows strong potential for ketone monitoring by offering enhanced sensitivity and accurate detection. However, variability in testing methods and BrAce cut-off levels poses challenges for comparing results, and a standardized methodology is urgently needed for clinical practice and widespread adoption. Respiratory status is a significant physiological indicator that represents the function and condition of an individual’s respiratory system [18]. Evaluating the partial pressure of CO_2_ (PCO_2_) is fundamental for accessing respiratory status. The gold standard for CO_2_ monitor is arterial blood gas (ABG) analysis [19,20]. However, this method is invasive, intermittent, and potentially painful [21]. Transcutaneous carbon dioxide (TcPCO_2_) monitoring provides a non-invasive alternative for monitoring PCO_2_ and is commonly used in sleep diagnostics, neonatal care, and exercise testing [22]. For continuous monitoring, a miniaturized and wearable device for TcPCO_2_ tracking is needed. To address this need, Ahmed et al. constructed a Metal–Organic Framework (MOF)-based sensor array for TcPCO_2_ monitoring (contribution 2). The MOF significantly enhanced the sensing performance due to its porous structure and large surface area. The system offered a broad detection range of 0–2% CO_2_ and high selectivity towards common volatile compounds in skin gases. Additionally, this sensor removed the need for external optical components by integrating the colorimetric sensing spots directly onto a lensless CMOS imager, reducing system size and enabling practical use in personal healthcare and exercise monitoring.

Autism Spectrum Disorder (ASD) is a neurodevelopmental condition that requires comprehensive management [23,24]. Individuals with ASD face challenges in social communications and exhibit unusual behavioral patterns, such as an intense focus on details [25,26]. As the prevalence of ASD has risen, there is an urgent need for effective diagnostic and management devices. Wearable sensors can serve as a valuable tool for continuous monitoring and for supporting interventions. Hernández-Capistrán et al. reviewed recent innovation and development of wearable sensors in ASD management (contribution 3). The authors categorized these devices by function, including eye-tracking devices, posture and balance sensors, physiological monitoring sensors, augmented reality technologies, and sleep-monitoring devices. Finally, they emphasized that building large-scale, real-world validation studies and increasing the number of FDA-cleared devices would enhance user acceptance of wearable sensors.

Cardiovascular diseases refer to a group of disorders affecting the heart and blood vessels [27,28]. Due to the rapid development of well-designed wearable sensors, cardiovascular diseases can now be effectively managed and monitored, realizing affordable at-home tracking. In contribution 4, Iqbal et al. reviewed recent advances in wearable devices for cardiovascular monitoring based on different technologies, such as galvanic contact, photoplethysmography (PPG), and radio frequency (RF) waves. The authors discussed recent wearable devices for detecting physiological indicators, namely electrocardiogram, heart rate, blood pressure, and thoracic fluid index. As for the future of cardiovascular device development, the authors suggested a closed-loop system encompassing diagnostic, monitoring, and prognostic functions. Other considerations, including the use of AI for real-time analysis and the development of self-powered cardiovascular devices, are also discussed.

Biofluids or biological body fluids contain components that are closely related to human ocular and systemic health status [29]. Tear-based wearable sensors are a non-invasive technology which focus on biomarker analysis (proteins, lipids, electrolytes, and metabolites) and facilitate early diagnosis of ophthalmological disorders, neurological diseases, and rental dysfunction [30]. A study by Rajan et al. reviewed recent technologies in tear-based wearable devices (contribution 5). This work provided a clear overview of tear composition and discussed various innovative approaches for biomarker analysis. The review highlighted the obstacles such as miniaturization, power consumption, and long-term stability. It also pointed out that integration with deep learning, AI, and the Internet of Things (IoT) could further enhance the potential of tear-based wearable sensors for monitoring and diagnosing. Sweat is another biofluid containing many important biomarkers, which reflect the physiological information, namely hydration state, fatigue, nutrition, and physiological changes [31]. Sweat is generated by glands, travels through ducts, and eventually reaches the epidermal surface via microscale pores [32]. This process enables the use of sweat sensors for non-invasive and real-time monitoring of sweat biomarkers. The bottleneck of wearable sweat sensing technology lies in analyzing sweat under low-perspiration conditions. In contribution 6, Konno et al. developed a wristwatch-style electrochemical biosensor that can continuously measure lactic acid (LA) from the skin in real time while minimizing the effects of sweat rate. By perfusing PBS across the skin with a miniaturized fluid control system to transport secretions to the sensor, the system preserves stable skin conditions and enables more reliable lactate monitoring. Additionally, this wristwatch system enables continuous lactate monitoring, demonstrating its potential for studying lactate secretion and distribution across various conditions.

Acquiring accurate physiological data during physical activity is essential to optimize training, prevent injuries, and support rehabilitation [33]. In contribution 7, Contreras-Briceño et al. studied the effect of high-flow nasal cannula (HFNC) in physical rehabilitation programs by assessing the oxygen saturation levels of accessory respiratory muscles (RMs). By using the NIRS wearable sensors, the researchers measured respiratory and leg muscle oxygenation during high-intensity cycling in 18 adults. HFNC significantly reduced respiratory muscle desaturation and hyperventilation compared to control, and participants reported less dyspnea, though leg fatigue and effort were unchanged. Overall, wearable optical biosensors demonstrated that HFNC can lower the cost of breathing during exercise, which supported its use in rehabilitation programs.

Advances in communication technology boost the continuous iteration and upgrading of wearable sensors [34]. Antennas are a key component of wearable sensors, which influence their portability, weight, flexibility, and overall communication performance [35]. Nanomaterials are currently being used in constructing antenna due to its exceptional conductivity, minuscule size, and good mechanical strength. In contribution 8, Wang and colleagues systematically review recent advances in nanomaterial-based wearable antennas across multiple dimensional scales, from 0-D nanoparticles to 1-D nanofibers and 2-D nanosheets. They show how materials such as metal nanoparticles, carbon nanotubes (CNTs), silver nanowires (AgNWs), and graphene enhance conductivity, reduce size and signal loss, improve radiation efficiency, and enable flexible, body-conforming designs. Their analysis demonstrates that nanomaterials can effectively address major challenges in wearable antenna design, including miniaturization, high performance, multi-band and multi-mode operation, and user safety and comfort.

This Special Issue highlights a diverse set of contributions that collectively demonstrate the remarkable progress in wearable biosensors for human health monitoring. Despite these advances, important challenges remain, including device miniaturization, achieving high accuracy and precision, and validating sensor performance in real-world applications. Continued progress in nanomaterials, detection strategies, system integration, artificial intelligence, and wireless communication technologies is expected to accelerate the development of next-generation wearable biosensors. As the field evolves, cross-disciplinary research with additive manufacturing (3D printing) [36,37] and the Internet of Things (IoT) [38,39] would play an important role for driving innovation and expanding the impact of wearable health monitoring systems.
